# Reversal of glucocorticoid resistance in Acute Lymphoblastic Leukemia cells by miR-145

**DOI:** 10.7717/peerj.9337

**Published:** 2020-06-16

**Authors:** Sili Long, Danwei Ren, Fangfang Zhong, Yana Niu, Xiang Qin, Dan Mu, Wenjun Liu

**Affiliations:** Department of Pediatrics, Affiliated Hospital of Southwest Medical University, Children’s Blood and Tumor PI laboratory, Birth Defects Clinical Medical Research Center of Sichuan Province, Luzhou, China

**Keywords:** Acute lymphoblastic leukemia, miR-145, Children, Glucocorticoid, Drug resistance

## Abstract

**Objective:**

To analyze the expression levels of miR-145 in ALL children and their effects on the prognosis of ALL and to explore the mechanism of miR-145 in reversing the resistance of ALL cells to glucocorticoids.

**Methods:**

A GEO database dataset was used to analyze the expression levels of miR-145 in ALL children. The association between miR-145 and childhood prognosis was analyzed by the TARGET database data. The expression levels of miR-145 in the glucocorticoid-resistant ALL cell line CEM-C1 were increased by lipofectamine 2000-mediated transfection. Cell proliferation inhibition experiments were performed to detect the effect of miR-145 on the response of CEM-C1 cell line to glucocorticoids. The expression levels of the apoptotic, autophagic and drug resistance-associated genes and proteins were detected by qPCR and western blot analysis.

**Results:**

The expression levels of miR-145 were decreased in ALL patients (*P* < 0.001) and the prognosis of ALL in children with high miR-145 expression was significantly improved (*P* < 0.001). Increased miR-145 expression can improve the sensitivity of CEM-C1 cells to glucocorticoids. The expression levels of the proapoptotic and the anti-apoptotic genes *Bax* and *Bcl-2* were increased and decreased, respectively, whereas the expression levels of the autophagicgenes *Beclin 1* and *LC* were increased. In addition, the expression levels of the drug resistance gene *MDR1* were decreased.

**Conclusion:**

The expression levels of miR-145 in ALL children were decreased and they were associated with disease prognosis. The data indicated that miR-145 can reverse cell resistance by regulating apoptosis of CEM-C1 cells and autophagy.

## Introduction

Acute lymphoblastic leukemia (ALL) is the most common malignant tumor in children and is associated with malignant proliferation of immature T or B lymphocytes (Pui et al., 2015). Glucocorticoids (GCs) are widely used in the treatment of ALL. These drugs induce apoptosis of the lymphoid progenitor cells. However, repeated use of GCs leads to drug resistance of tumor cells, resulting in treatment failure or recurrence. It has been reported that 20% of ALL children are resistant to GCs and that the proportion of GC resistance in recurrent ALL children can reach 70% (Xie et al., 2019). The low reactivity to the prednisone-induced test is also one of the main indicators of the increase in the risk of relapse and treatment failure of childhood ALL (Hematology Section, Chinese Academy of Pediatrics, Editorial Committee, 2014). It has been shown that GCs play a critical role in the treatment of ALL. Therefore, the identification of the mechanism of GC resistance and the development of new treatment strategies can fully unlock the therapeutic potential of GC and significantly improve the prognosis of ALL.

MicroRNAs (MiRNAs) are small non-coding single-stranded RNAs containing approximately 22 nucleotides that display regulatory functions against mRNAs after transcription. They also participate in the regulation of GC sensitivity through a variety of mechanisms and are involved notably in the regulation of the intracellular expression of the GC receptors. The sensitivity of tumors to GCs may be affected by miRNAs that can be used as biomarkers or can provide potential strategies for overcoming drug resistance (Wang et al., 2017). A previous study demonstrated that miR-145 exhibited low expression levels in adult T-ALL and that it was significantly associated with the deterioration of the patient health condition (Xia et al., 2014). Therefore, miR-145 may become a prognostic marker and potential therapeutic target for ALL patients (Xia et al., 2014). The present study analyzed the expression levels of miR-145 in childhood ALL and explored the correlation between miR-145 and glucocorticoid resistance in ALL cells. The antitumor effect of miR-145 was examined in the glucocorticoid-resistant ALL cell line CEM. The mechanism by which C1 affected the sensitivity to glucocorticoids was also explored.

## Data and Methods

### Dataset

ALL samples from children were collected. The miRNA expression profile dataset GSE56489 was downloaded from the NCBI’s GEO database (https://www.ncbi.nlm.nih.gov/geo/). This dataset contained 43 bone marrow samples from ALL children and 14 age-matched healthy control samples, including 21 males and 22 females, and the average age is 6.8 ± 4.5 years. The data were processed using the GPL14132 dataset and the *Homo sapiens* miRBase 15.0 annotation (Duyu et al., 2014). In addition, the miRNA expression profiles of childhood ALL were downloaded from the TARGET database (Therapeutically Applicable Research To Generate Effective Treatments, https://ocg.cancer.gov/programs/target) (Lu et al., 2019) managed by the NCI’s Office of Cancer Genomics and Cancer Therapy Evaluation Program. The datasets and clinical data were divided into high and low expression groups according to the median gene expression level. The clinical data of the children were combined to further analyze the miRNAs associated with the prognosis of the children.

### Cell culture and transfection

The CEM-C1 cell line is a human acute T-lymphocytic leukemia (T-ALL) cell line resistant to dexamethasone (DEX). This cell line was donated by Professor Ma Zhigui (Children’s Hematology and Oncology Department of West China Second Hospital of the Sichuan University) (Yan et al., 2014). The cells were cultured in an RPMI-1640 medium containing 100 μg/ml penicillin G, 100 μg/ml streptomycin and 10% fetal bovine serum in an incubator at 37 °C, in the presence of 5% CO_2_. When the cells were confluent to 85% or more, the cells were prepared for subculture. Following washing, CEM-C1 cells were grown to the log phase and seeded in a 6-well plate in the presence of serum-free and antibiotic-free culture medium. miR-145 mimic and miR-145 mimic NC control were transfected with Lipofectamine 2000 transfection reagent according to the manufacturer’s instructions. The miR-145 inhibitor and the miR NC control sequences were transfected into the CEM-C1 cells. They were divided into the following groups: miR-145 mimic: MM, miR-145 mimic NC: MMN, miR-145 inhibitor: MI, miR-145 inhibitor NC: MIN. Four groups were used for backup experiments at 48 h following transfection. A transfected fluorescent miR-145 mimic was used to observe cellular morphology by fluorescence microscopy and to rapidly detect cell transfection efficiency.

### qRT-PCR detection of miR-145 and associated-gene expression in each group of cells

The aforementioned groups of cells were collected and total RNA was extracted by the total RNA extraction kit and reverse-transcribed into cDNA. The target genes, miR-145 and the internal reference gene *GAPDH*, *RNU6B* were amplified by fluorescent quantitative PCR. The sequences of the primers of each gene are shown in Table 1. The cDNA samples were pre-denatured at 95 °C for 60 s, denatured at 95 °C for 15 s, annealed at 60 °C for 15 s and extended at 72 °C for 45 s. A total of 40 cycles were used for fluorescent quantitative PCR reaction conditions. The data were analyzed by the formula: 2^−ΔΔCt^, indicating the relative expression levels of the target gene mRNA.

**Table 1:** Primer sequences for related mRNAs.

	F/R	Sequence
miRNA-145	F	GTCCAGTTTTCCCAGGAATCCCT
	R	CAGGTCAAAAGGGTCCTTAGGGA
*RNU6B*	F	CGCAAGGATGACACGCAAATTCGTGAAGCGTTCCATATTTTT
	R	GCGTTCCTAGTGTGCGTTTAAGCACTTCGCAAGGTATAAAAA
*GAPDH*	F	CAATGACCCCTTCATTGACC
	R	GACAAGCTTCCCGTTCTCAG
*Bax*	F	GACGAACTGGACAGTAACATGGA
	R	GCAAAGTAGAAAAGGGCGACA
*Bcl-2*	F	CCCGTTGCTTTTCCTCTGG
	R	ATCCCACTCGTAGCCCCTCT
*LC-I*	F	CCAGCCGACCGCTGTAAG
	R	TCATGTTGACATGGTCCGGG
*LC-II*	F	CAGCGTCTCCACACCAATCT
	R	TCTCCTGGGAGGCATAGACC
*Beclin 1*	F	CGTGTCACCATCCAGGAACT
	R	TCTCCAAACAGCGTCTGGCT
*MDR1*	F	CCAGAAACAACGCATTGCCA
	R	GTGCCATGCTCCTTGACTCT

### CCK8 detects miR-145 affects CEM-C1 cell responsiveness to dexamethasone

Following transfection, CEM-C1 cells were incubated for 48 h and the cell density was adjusted to 1 × 10^5^ cells per well. The cells were seeded in 96-well plates, and 20, 40, 80, 160 and 320 μg/ml dexamethasone (DEX) were added to each group, respectively. A total of 3 replicates were set up for each group of cells and the experiment was repeated 3 times. Following 48 h of incubation, 10 μl CCK8 reagent was added to each well. The samples were incubated in a CO_2_ incubator for 4 h and the OD value of each well was measured by the absorbance (A) reading at 450 nm using a multifunctional microplate reader. Calculated according to the formula: cell proliferation inhibition rate = (OD_control_−OD_experimental_)/(OD_control_−OD_blank_) ×100%; IC_50_ = Ig−1 {Xm−I (∑p−0.5)}; Resistance Index (RI) = IC_50__control_/IC_50__experimental_.

### Flow cytometry for miR-145 detection of CEM-C1 cell apoptosis

CEM-C1 cells were collected 48 h following transfection, washed with pre-cooled PBS, and mixed with 500 μl binding Buffer. A total of 100 μl of cell suspension was used for sub-assembly. Each sample was mixed with 5 μl Annexin-V FI/TC and 5 μl PI staining solution and subsequently incubated for 15 min at room temperature in the dark. A total of 400 μl of binding buffer was added and 1 × 10^4^ cells were analyzed by flow cytometry. Annexin-V FI/TC positive cells were classified as progressive apoptotic cells, while FI/TC negative cells were classified as viable cells.

### Acridine orange staining indicates the effects of miR-145 on the induction of CEM-C1 cell apoptosis

CEM-C1 cells were collected 48 h following transfection, washed twice with PBS and added to a final concentration of 100 μg/ml acridine orange stain. The cells were stained for 30 min in the dark and observed under a fluorescence microscope.

### Western blot analysis of apoptotic and autophagic proteins in CEM-C1 cells

CEM-C1 cells were collected at 48 h following transfection and total cell proteins were extracted according to the instructions of the whole protein extraction kit. The protein concentration was measured by the BCA method and the same amount of protein was used for each sample. The protein samples were boiled for 5 min in 5 × SDS loading buffer. Following SDS-PAGE electrophoresis, the proteins were transferred to PVDF membranes and blocked with 5% skimmed milk powder for 2 h. The samples were rinsed thoroughly with TBST (10 min, 3 times). Bax, Bcl-2, LC3 I/II, Beclin-1 and MDR1 polyclonal antibodies were diluted at a 1:1,000 dilution ratio and the internal reference protein GAPDH was diluted at a 1:10,000 ratio. The samples were incubated overnight at 4 °C, washed thoroughly with TBST and goat anti-rabbit secondary antibody was added at a 1:2,000 dilution ratio. The samples were further incubated at room temperature for 1 h and washed thoroughly with TBST (10 min, 3 times). Finally, ECL luminescence reagents were added for protein detection. The images were analyzed by the Gel-Pro analyzer software and the relative expression levels of the target proteins were expressed as the relative ratio of the expression of each protein band to the relative expression of the GAPDH protein band.

### Statistical analysis

Statistical analysis of the data was performed using the GraphPad Prism 7.0 software. The experimental data were expressed as mean±standard deviation (mean ± SD). The data of multiple groups were compared by one-way analysis of variance. The data of the two groups were compared by the *t* test. The difference was statistically significant at *P* < 0.05.

## Results

### Association between miR-145 and childhood ALL

#### Expression levels of miR-145 in ALL samples from children

**Figure 1:**
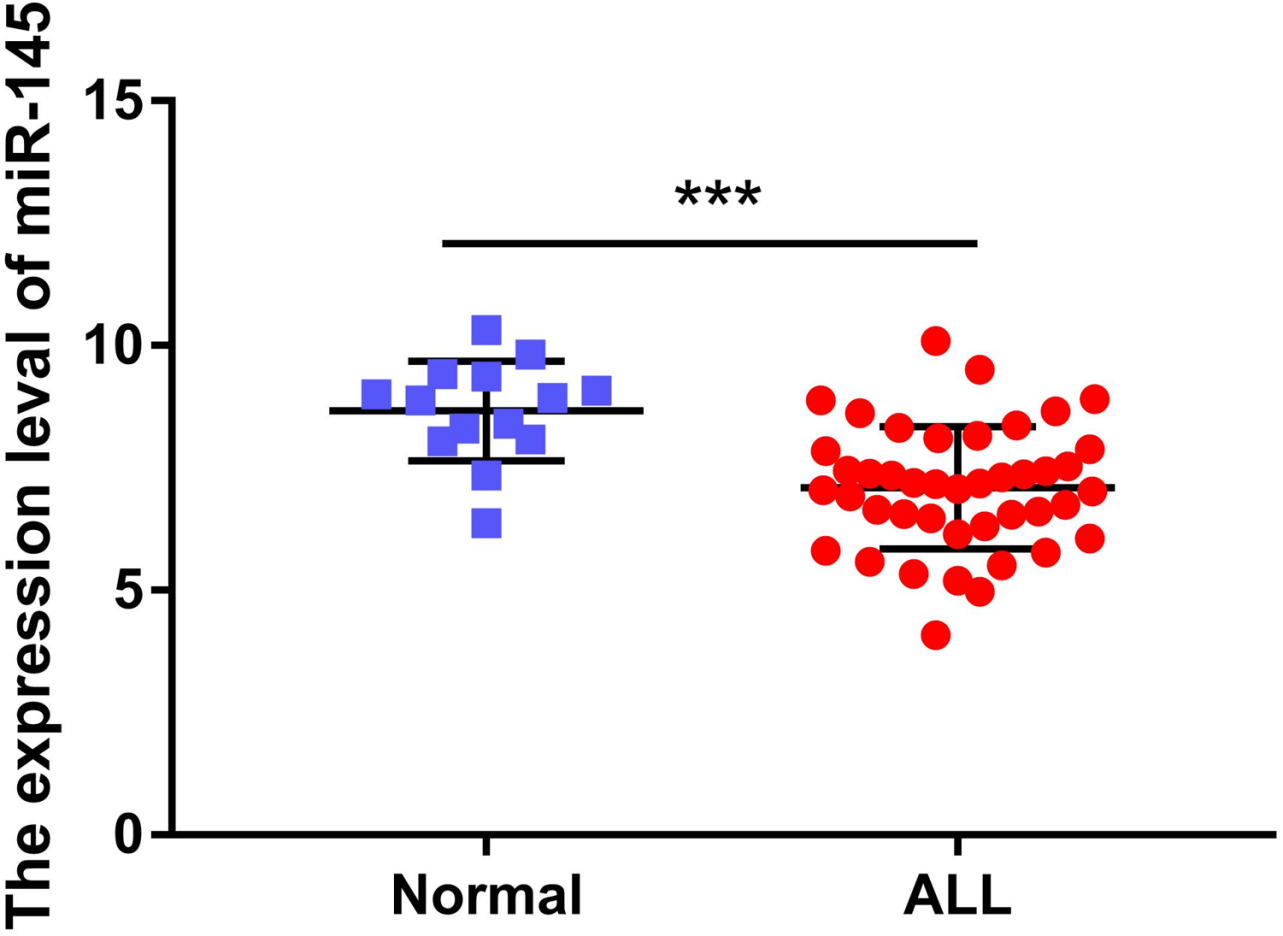
Comparison of the expression levels of miR-145 in ALL bone marrow samples and bone marrow samples from age-matched healthy control samples (data from Duyu et al. (2014)). ***P < 0.001.

The expression levels of miR-145 in the bone marrow samples of 43 children with ALL and in the corresponding samples of 14 healthy children were determined. The expression levels of miR-145 in ALL children and in age-matched healthy control samples were 7.09 ± 0.19 and 8.67 ± 0.27, respectively. The expression levels of miR-145 in children with ALL were significantly lower than those of the healthy subjects (*t* = 4.26, *P* < 0.0001) (Fig. 1).

#### Association between miR-145 levels and prognosis of children with ALL

**Figure 2:**
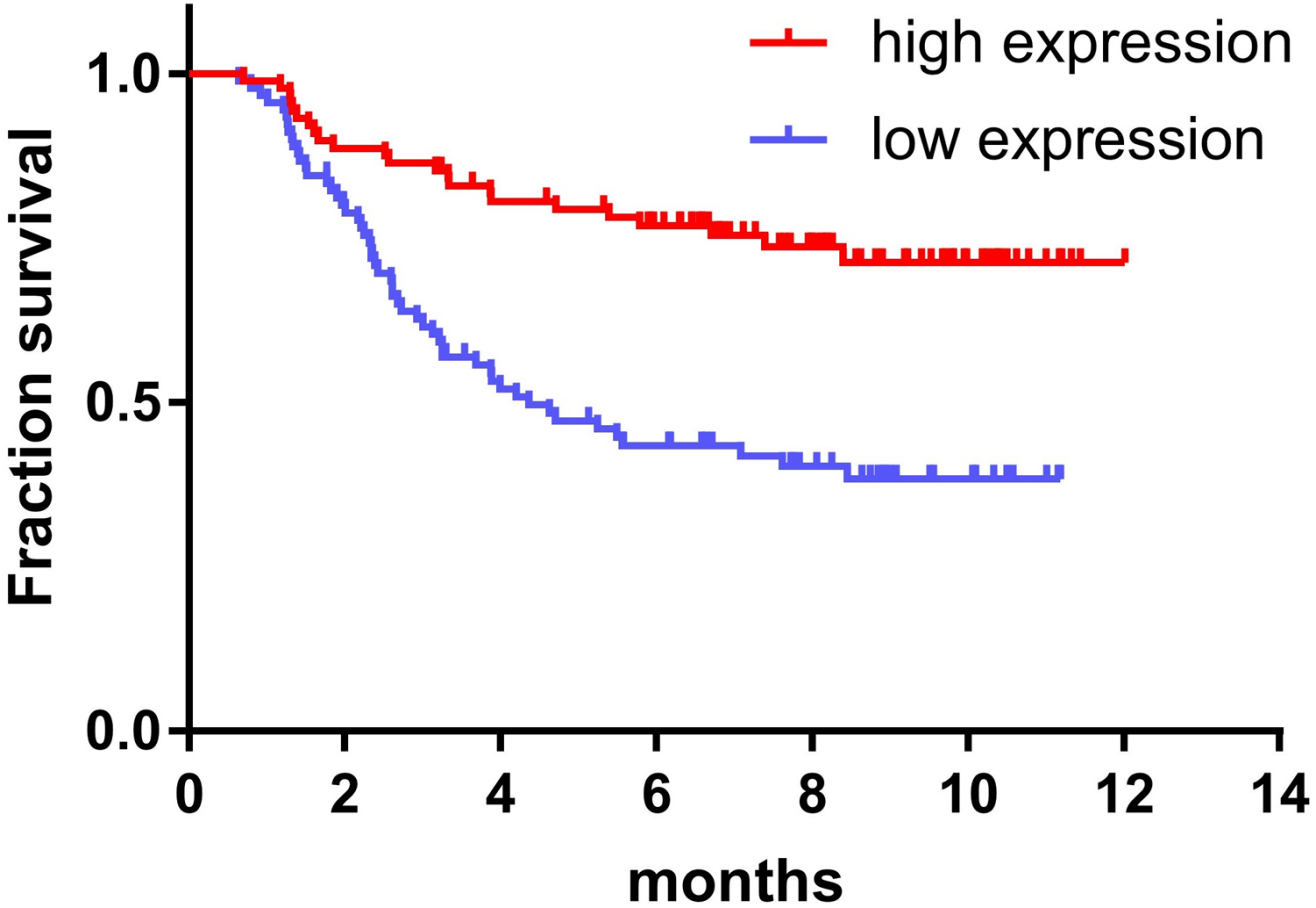
Relationship between miR-145 expression and prognosis in children with ALL.

The expression of miR-145 in bone marrow samples of 179 children with ALL was determined using the TARGET database and the log-rank survival analysis. These results were combined with clinical data showing that the prognosis of children with ALL in the high expression miR-145 group was significantly higher than that of the low expression group (*P* < 0.001) (Fig. 2).

### Expression levels of miR-145 in CEM-C1 cells

#### Transfection of miR-145 as determined by fluorescence microscopy

**Figure 3:**
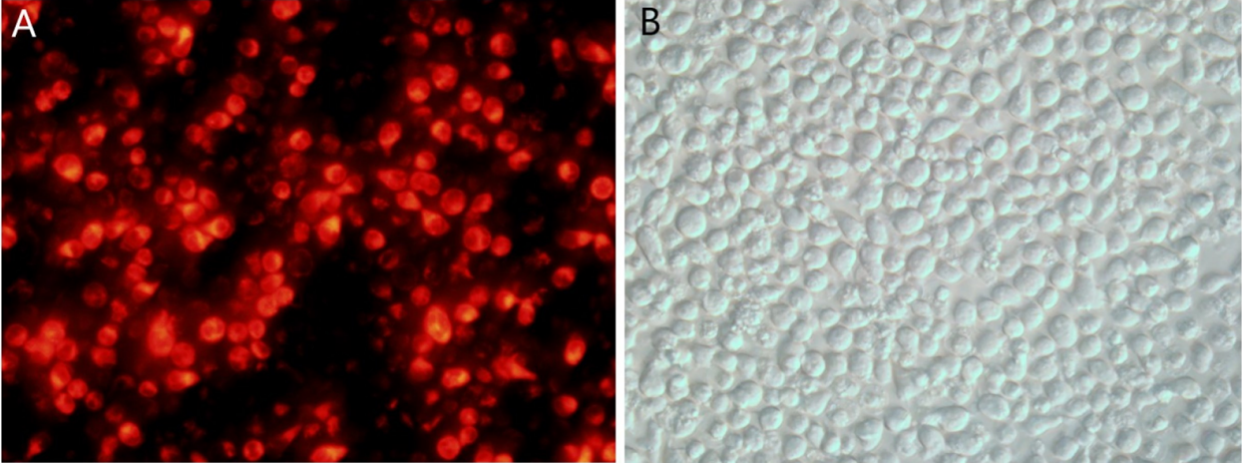
CEM-C1 cell transfection using fluorescent miR-145 mimic. (A) Fluoroscopy, red fluorescence indicated miR-145 mimic transfer into the cells. (B) Same field of view, cell morphology under optical microscopy investigation.

Following transfection of CEM-C1 cells with fluorescent miR-145 mimics, they were observed using fluorescence microscopy. The transfected cells appeared red under the fluorescence microscope, as shown in Fig. 3A. The cell morphology was also observed using light microscopy (Fig. 3B). The efficiency of transfection was estimated to approximately 70% and a small amount of cells were not viable due to the toxicity of the transfection reagent.

#### qPCR detection of transfection efficiency of each group of cells

**Figure 4:**
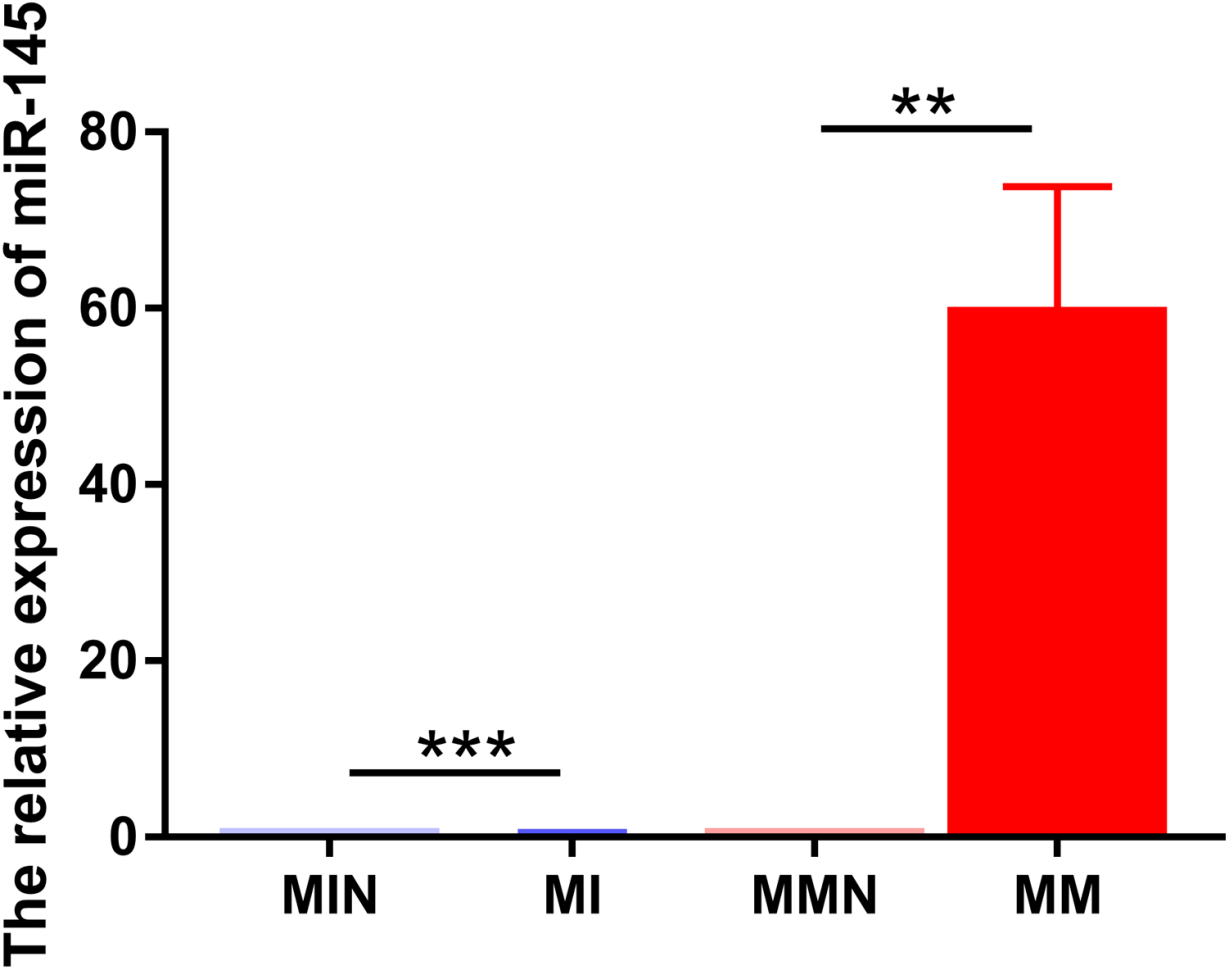
Relative expression levels of miR-145 in each group of CEM-C1 cells following transfection. **P < 0.01, ***P < 0.001.

The same method was used to transfect miR-145 mimic, miR-145 mimic NC negative control, miR-145 inhibitor and miR-145 inhibitor NC negative control sequences into CEM-C1 cells for 48 h. Subsequently, miR-145 expression was detected in the four groups of cells by qPCR. The expression levels in the medium were further investigated in order to assess successful transfection. miR-145 exhibited significantly higher expression in the MM group (*P* < 0.01), whereas its expression levels were significantly decreased in the MI group (*P* < 0.001) (Fig. 4).

### miR-145 improves sensitivity of CEM-C1 cells to Dexamethasone

**Figure 5:**
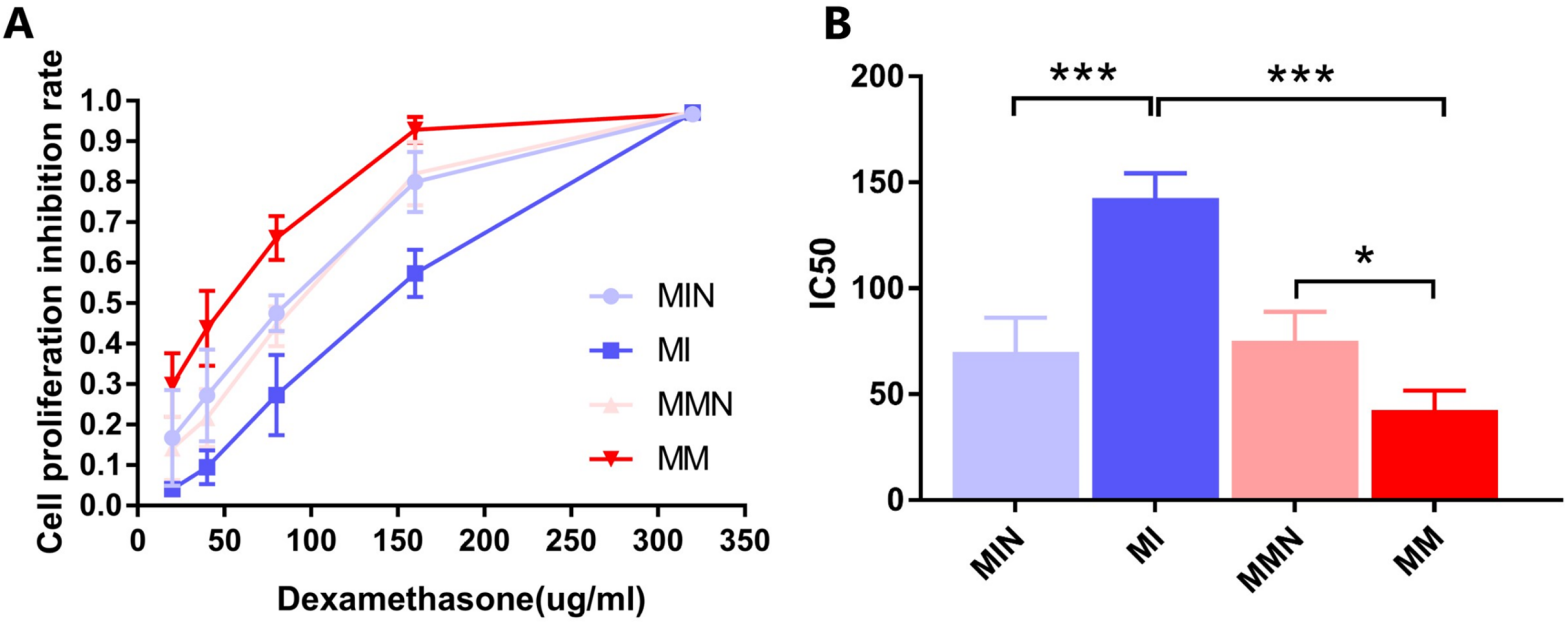
Effects of dexamethasone on CEM-C1 cells in each group. (A) Effects of different concentrations of dexamethasone on the proliferation inhibition rate of cells in each group. (B) Statistics of IC50 of each group of cells. *P < 0.05, ***P < 0.001.

The results indicated that DEX exhibited a significant inhibitory effect on the cells of each group (Fig. 5A). The IC_50_ values of the MIN, MI, MMN and MM groups were (69.94 ± 9.33), (142.60 ± 6.74), (75.24 ± 7.86), ( 42.66 ± 5.26) μg/ml, compared with the IC_50_ of each group. Significant differences were noted using *t* test analysis was statistically significant (Fig. 5B); The resistance index (RI) was 3.34 and was estimated by the IC_50_ measurement of the MM and the MI groups.

### Effects of miR-145 on the induction of apoptosis of CEM-C1 cells

#### Flow cytometry for detection of the apoptotic effects of miR-145 in each group

**Figure 6:**
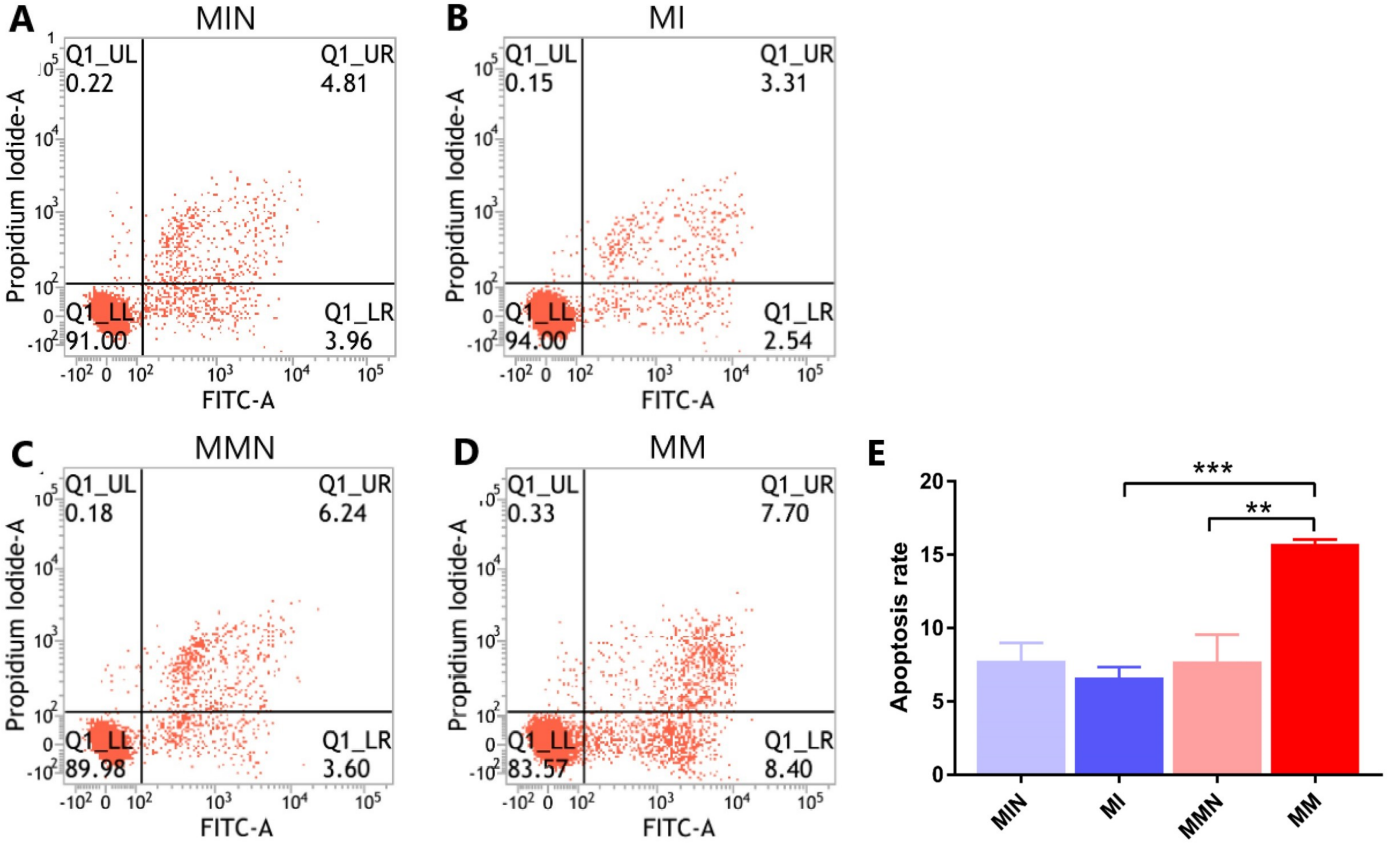
The effects of miR-145 on apoptosis of CEM-C1 cells in each group. (A) The apoptotic rate of the MIN group was 8.77%. (B) The apoptotic rate of the MI group was 5.85%. (C) The apoptotic rate of the MMN group was 9.84%. (D) The apoptotic rate of the MM group was 16.10%. (E) The comparison of the apoptotic rates of each group revealed significant differences. ** *P* < 0.01, *** *P* < 0.001.

Following 48 h of transfection, the apoptotic rate of the MIN group (7.80 ± 0.70%) was lower than the apoptotic rate of the MI group (6.65 ± 0.40%). The apoptotic rate of the cells in the MMN group was 7.73 ± 1.06%, whereas the apoptotic rate of the cells of the MM group was 15.76 ± 0.17%. The apoptotic rate of the drug-resistant cell lines with increased expression of miR-145 was significantly increased (*t* = 7.45, *P* < 0.01) (Fig. 6).

#### Effects of miR-145 on the expression levels of the apoptotic genes *Bax*, *Bcl-2*, *MDR1* and of their corresponding proteins

**Figure 7:**
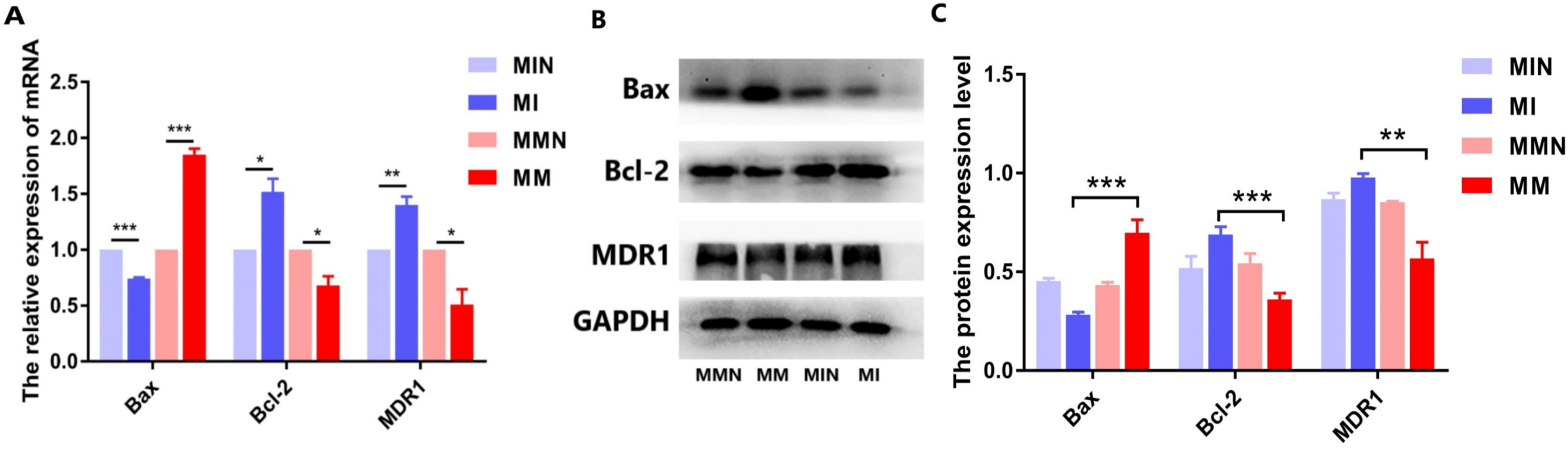
The effects of the miR-145 expression on the expression levels of the Bax, Bcl-2 and MDR1 proteins in CEM-C1 cells. (A) The expression levels of the *Bax*, *Bcl-2*and *MDR1*genes were compared in each group. (B) Western blot analysis was used to detect the expression levels of the aforementioned markers in each group of cells. The expression levels of Bax, Bcl-2 and MDR1 proteins. (C) The comparison of the expression levels of Bax, Bcl-2 and MDR1 proteins in each group of cells. *P < 0.05, **P < 0.01, ***P < 0.001.

Following qPCR analysis, the expression levels of the pro-apoptotic gene *Bax* in the MM group were higher than those of the MI group (*P* < 0.01). The expression levels of the anti-apoptotic gene *Bcl-2* were decreased in the MM group (*P* < 0.01). The expression levels of the drug resistance gene *MDR1* were also decreased in the MM group (*P* < 0.01) (Fig. 7A). The results of the western blot analysis were consistent with the qPCR results and the expression levels of the Bax protein were higher than those noted in the MM group (*P* < 0.001). The expression levels of the Bcl-2 protein in the MM group were decreased (*P* < 0.01), whereas the expression levels of the MDR1 protein in the MM group were also significantly decreased (*P* < 0.001) (Figs. 7B and 7C).

### Effects of miR-145 on the induction of autophagy in CEM-C1 cells

#### Acridine orange detection of miR-145-mediated autophagy in each group of cells

**Figure 8:**
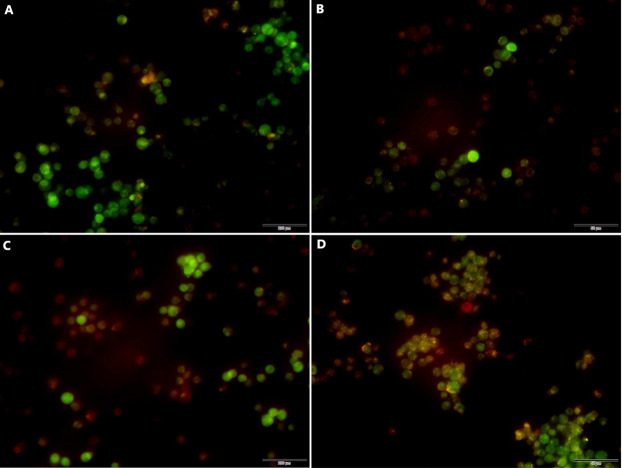
Acridine orange staining indicating the induction of autophagy by miR-145 in CEM-C1 cells. (A) A small amount of orange autophagic vesicles in the MIN group. (B) Absence of orange autophagic vesicles in the MI group. (C) A small number of orange autophagic vesicles in the MMN group as determined by orange-red fluorescence. (D) The orange-red fluorescence in the MM group was significantly increased, indicating that the number of autophagic vesicles was increased and that the number of cells containing acidic vesicles was also increased.

Following transfection the cells were cultured for 48 h and the effects of miR-145 on the induction of autophagy of the drug-resistant cells were detected by acridine orange staining. The data indicated a higher number of red-spotted acidic vesicles (autophagosomes) in the cytoplasm of the MM group. Moreover, the data indicated that increased expression levels of miR-145 could promote autophagy in drug-resistant cells (Fig. 8).

#### Effects of miR-145 on the expression of the drug resistance gene *MDR1*, of the autophagic genes *LC* and *Beclin 1* and of their corresponding proteins in each group

**Figure 9:**
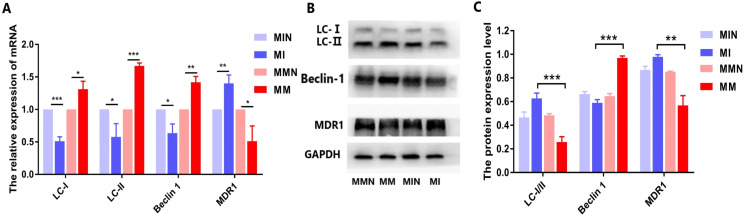
Effects of miR-145 on LC-I, LC-II, Beclin 1, MDR1 gene and protein expression in each group. (A) *LC-I*, *LC-II*, *Beclin 1*, *MDR1* gene expression in each group. (B) Western blot detection. The expression levels of LC-I, LC-II, Beclin 1 and MDR1 protein in each cell group of cells. (C) The expression levels of LC-I/II, Beclin 1 and MDR1 proteins in each cell group were compared. *P < 0.05, **P < 0.01, ***P < 0.001.

When autophagy is induced, cytosolic LC-I is enzymatically converted to LC-II, and this process can be evaluated based on the ratio of LC-I/II. qPCR analysis demonstrated that the expression levels of the autophagy-associated genes *LC* and *Beclin 1* in the MM group were higher than those in the MI group, while the ratio of LC-I/II in the MM group was reduced (Fig. 9A). All these differences in the results were statistically significant (Fig. 9A). The western blot data were also consistent with the qPCR results. In the MM group, the LC-I/II ratio was decreased (*P* < 0.05) and the levels of the autophagic protein Beclin 1 were increased (*P* < 0.01). In addition, cyclin B1 expression was also significantly increased (*P* < 0.001) (Figs. 9B and 9C).

**Figure 10:**
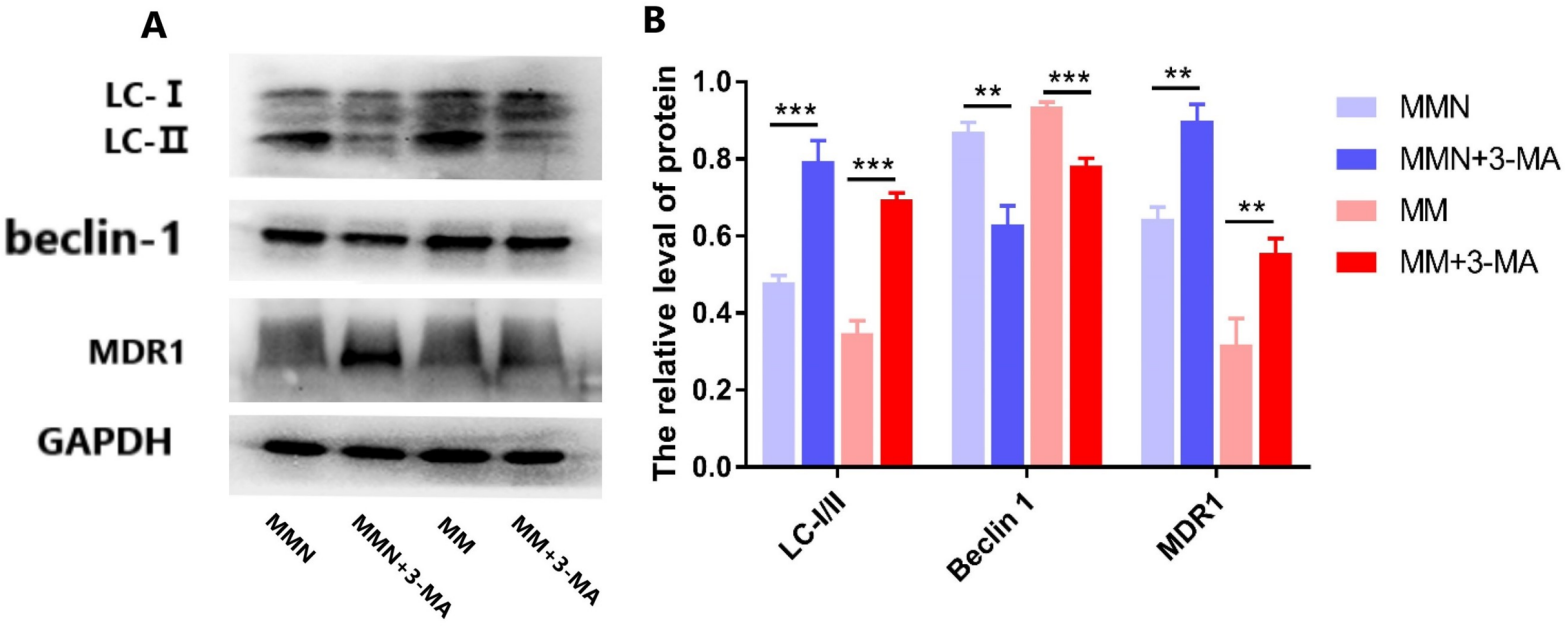
Effect of autophagy inhibitor on miR-145-mediated resistance of ALL cells to glucocorticoid. (A) Western blot detection. The expression levels of LC-I, LC-II, Beclin 1 and MDR1 protein in each cell group of cells. (B) The expression levels of LC-I/II, Beclin 1 and MDR1 proteins in each cell group were compared. **P < 0.01, ***P < 0.001.

The autophagy inhibitor 3-Methyladenine (3-MA) was added to the MM and MMN groups in order to confirm whether autophagy-related genes interact with miR-145 to improve the resistance of CEM-C1 cells to glucocorticoids. The results indicated that following addition of the inhibitor, the ratio of the autophagic protein LC-I/II increased significantly, whereas the expression levels of Beclin 1 were decreased and the expression levels of the drug resistance protein MDR1 were increased following addition of the inhibitor (Fig. 10).

## Discussion

Glucocorticoids (GCs) can induce apoptosis of ALL cells and are the main chemotherapeutic drugs used in children with ALL. The resistance of patients with ALL to GCs is also a major challenge in clinical treatment (Pui et al., 2012). MicroRNAs (miRNAs) are a group of small non-coding RNAs that are 22 nucleotides in length. Accumulating evidence has suggested that miRNAs play an important role in several biological processes and that their main role is to bind to the 3′-UTR region of the target sequence, resulting in inhibition or degradation of mRNA molecules (Iorio & Croce, 2017). miR-145 is located on chromosome 5 (q32–33) and is a typical fragile site of the human genome. The present study demonstrated that in a variety of tumors, such as glioma and ovarian, lung and esophageal cancers, the expression levels of miR-145 were lower than those of the adjacent or normal tissues, indicating that miR-145 plays a role in tumor suppression (He et al., 2019; Hua et al., 2019; Li et al., 2019; Zheng et al., 2019). Moreover, miR-145 may be involved in ALL resistance to GCs (Bhadri, Trahair & Lock, 2012). However, no specific mechanism of action has been discovered to date. In the present study, we found that the expression levels of miR-145 in childhood acute lymphoblastic leukemia were lower than those noted in healthy controls, suggesting that miR-145 played a regulatory role in the development of childhood ALL (Fig. 1). In addition, it was found that the survival time of children with high expression of miR-145 was significantly higher than that of the low expression group, further indicating that miR-145 was associated with the prognosis of children with ALL (Fig. 2). The adaptation of the chemotherapeutic dose and schedule of GC administration according to the tumor resistance has become a new focus for the diagnosis and treatment of ALL children. Therefore, it is possible to achieve a precise treatment plan for children with ALL.

At the cellular level, the present study demonstrated increased expression levels of miR-145 in the GCs-resistant ALL cell line CEM-C1 by transfecting miR-145 mimic and miR-145 inhibitor sequences to the cells. A specific cell line with increased expression of miR-145 was identified. The proportion of cells undergoing apoptosis and autophagy was significantly higher than that of the low expression group (Figs. 5 and 6), which confirmed the aforementioned hypothesis. Apoptosis is a highly ordered and active death process regulated by specific genes, which is considered one of the main ways to regulate body homeostasis. The Bcl-2 protein family plays a “main switch” role in the process of apoptosis by regulating the opening and closing of mitochondrial PT pores (Agrawal et al., 2011). Bcl-2 is an anti-apoptotic gene initially identified in follicular lymphoma, which inhibits apoptosis via a calcium-dependent specific protein phosphorylation mechanism (Preisler & Gopal, 1994). This function is considered one of the main mechanisms responsible for drug resistance (Preisler & Gopal, 1994). Bax is the most widely studied pro-apoptotic protein in the Bcl-2 family. It is localized in the cytoplasm and promotes the opening of PT pores, releasing cytochrome C, activating caspase-9, and activating the mitochondrial apoptotic pathways (Preisler & Gopal, 1994). In addition, it antagonizes the Bcl-2 anti-apoptotic effect and accelerates cell death (Perkins et al., 2000). Autophagy is a physiological process in which cells self-degrade. This process has an important function in maintaining internal cellular stability. Following induction of autophagy in the cells, a protective effect is conferred from cell hazards, such as harsh environment and type II programed death or autophagic death, which is different than apoptosis (Jia et al., 2009; Cheallaigh et al., 2011; Park & Cuervo, 2013). Among these associated genes, *Beclin 1* is involved in the initiation of autophagy. This protein is located in the autophagosome membrane. Its expression is positively associated with the levels of autophagy and plays an important role in its induction. The increase in the expression levels of these markers is an important and reliable indicator of autophagy (Luo & Rubinsztein, 2010; Russell et al., 2013). LC3 is a homolog of the autophagy-related gene (ATG8) gene in mammalian cells. It is targeted to the autophagosome membrane and participates in the induction of autophagy. LC-I is localized in autophagosomes and is cleaved into LC-II by lysosomes. Therefore, the decrease in the LC-I/II ratio is the hallmark of autophagy (Mizushima, Yoshimori & Ohsumi, 2003).

In the present study, qPCR and western blot analyses indicated that miR-145 could promote apoptosis by upregulating the levels of the anti-apoptotic protein Bax and by downregulating the levels of the pro-apoptotic protein Bcl-2. Moreover, miR-145 promoted cell self-regulation by controlling the levels of the autophagic proteins LC and Beclin 1. The expression levels of the resistance protein MDR1 were also decreased following the increase of miR-145, indicating that miR-145 could improve the resistance of the CEM-C1 cell line to GCs (Figs. 5–8). The drug resistance mechanism of the ALL cell line CEM-C1 to GC treatment was not fully detailed and further experiments are required to explore the effects of miR-145 on other signaling pathways and key signaling proteins. Additional in-depth mechanistic analysis is required by animal studies and clinical trials.

## Conclusions

In summary, the present study indicated that miR-145 expression was downregulated in ALL children and that it was associated with disease prognosis. miR-145 can increase the sensitivity of CEM-C1 cells to GCs by promoting the induction of autophagy and apoptosis. These data may provide a novel avenues for the clinical diagnosis and treatment of children with ALL.

## Supplemental Information

10.7717/peerj.9337/supp-1 Supplemental Information 1 Figure 4-7 Raw data.

10.7717/peerj.9337/supp-2 Supplemental Information 2 Figure 9,10 Raw data.

10.7717/peerj.9337/supp-3 Supplemental Information 3 TARGET dataset.

## Additional Information and Declarations

### Data Availability

The following information was supplied regarding data availability: Data is available at NCBI GEO: GSE56489. The raw data is available in the Supplemental Files and at TARGET Data Matrix (https://ocg.cancer.gov/programs/target/data-matrix) under Disease heading: “ALL Phase 2 (B-ALL, T-ALL, Dicentric, Xenograft)” and miRNA heading: “miRNA-DCC Open*”.
