# Supplementary figures and images for: Reversal of glucocorticoid resistance in Acute Lymphoblastic Leukemia cells by miR-145
Source: PeerJ. 2020 Jun 16;8:e9337. doi: 10.7717/peerj.9337 (PMC7304417; doi:10.7717/peerj.9337)

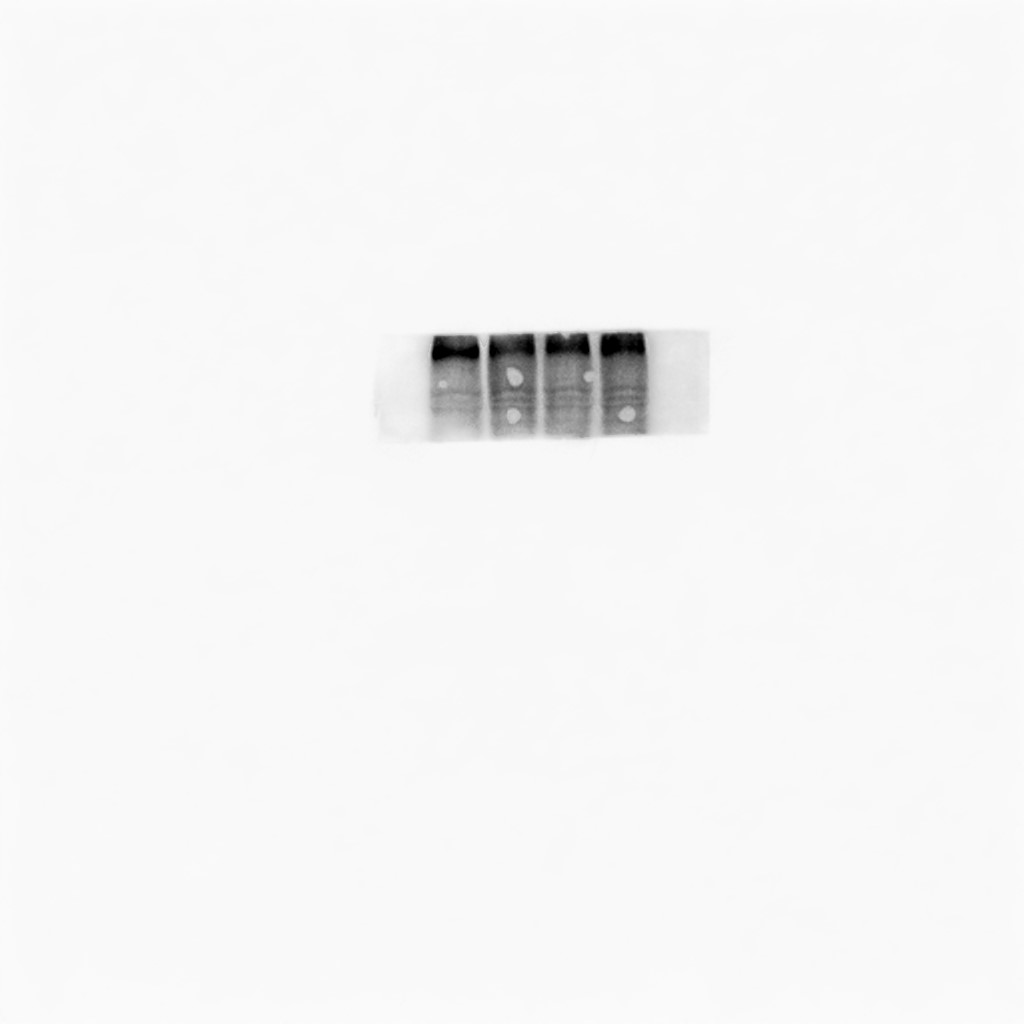

Supplement: Supplemental Information 1 [file peerj-08-9337-s001.zip › 7b/MDR1/MDR1 11.14.Tif]

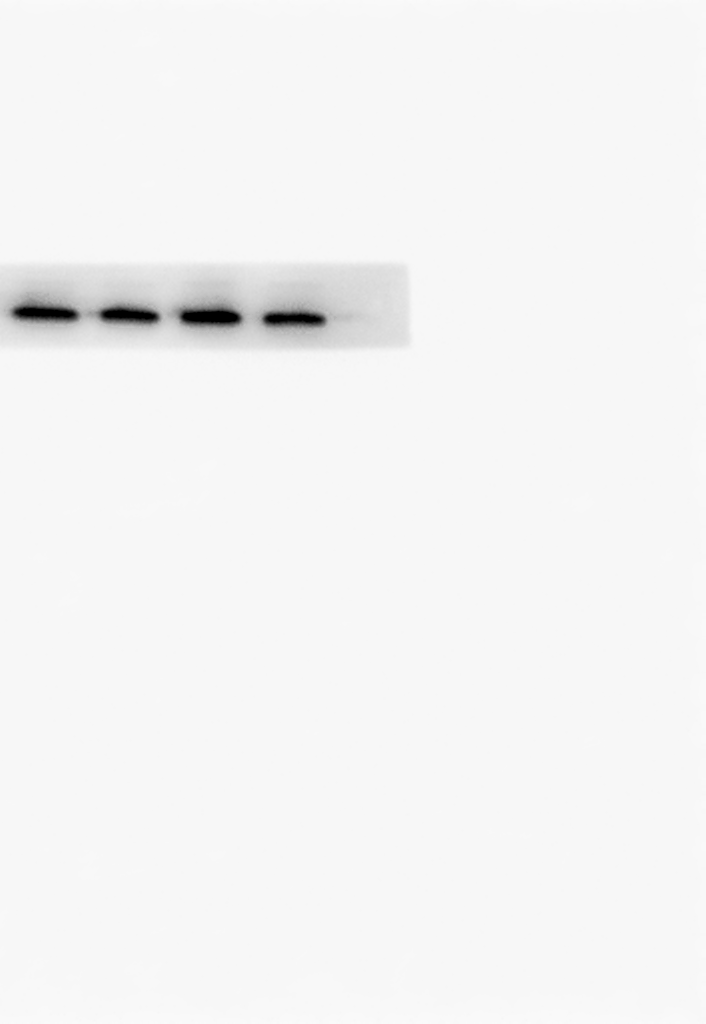

Supplement: Supplemental Information 2 [file peerj-08-9337-s002.zip › 9b/LC/GAPDH 1.2.Tif]

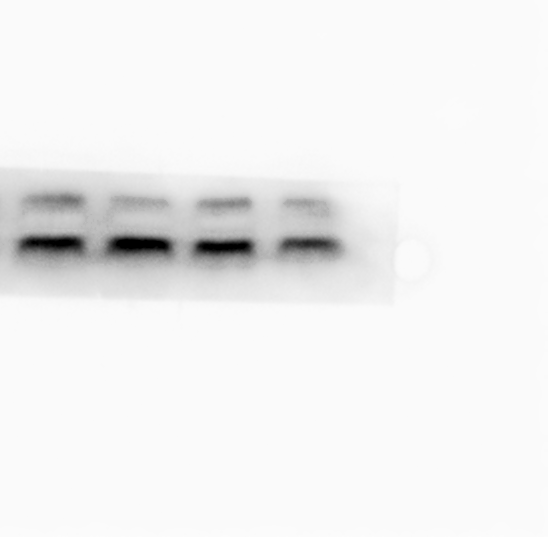

Supplement: Supplemental Information 2 [file peerj-08-9337-s002.zip › 9b/LC/LC 1.2.Tif]

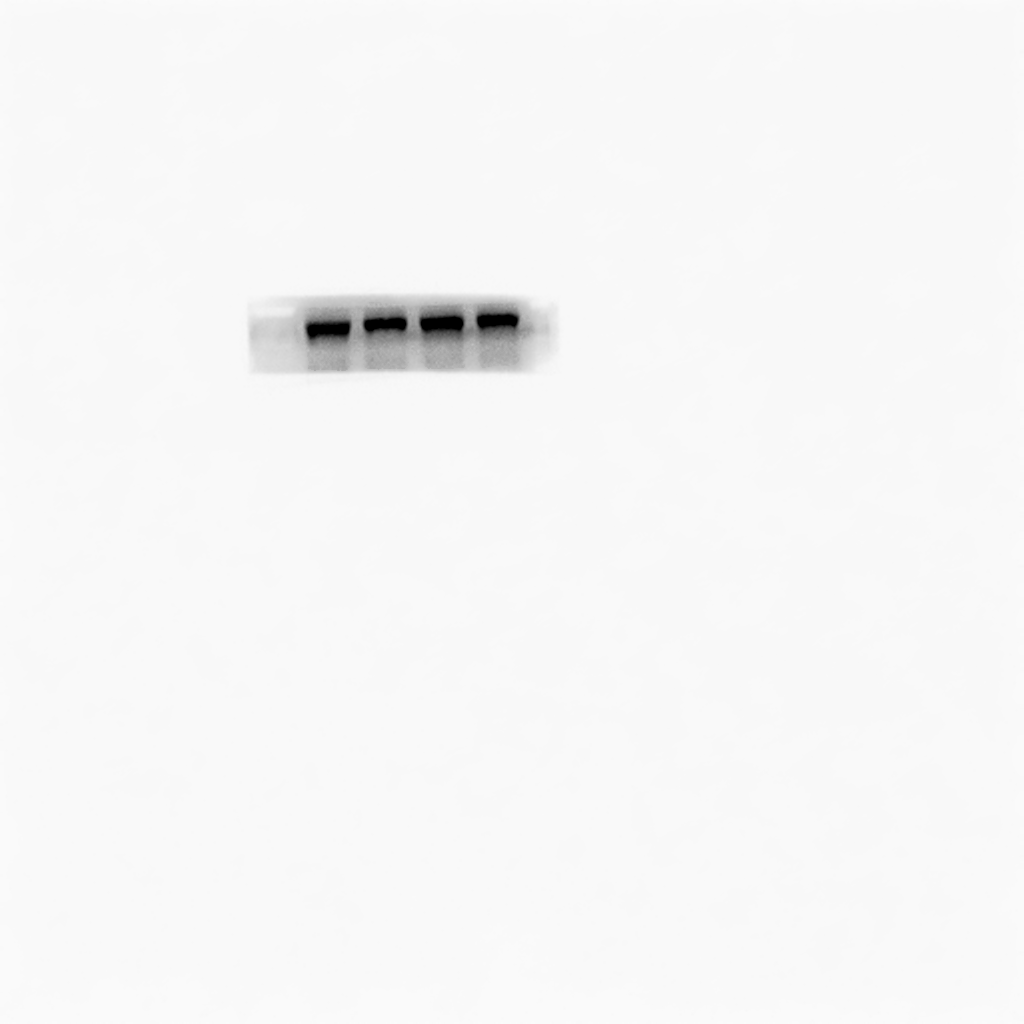

Supplement: Supplemental Information 2 [file peerj-08-9337-s002.zip › Figure 10/Beclin-1/BECLIN 3.19.Tif]

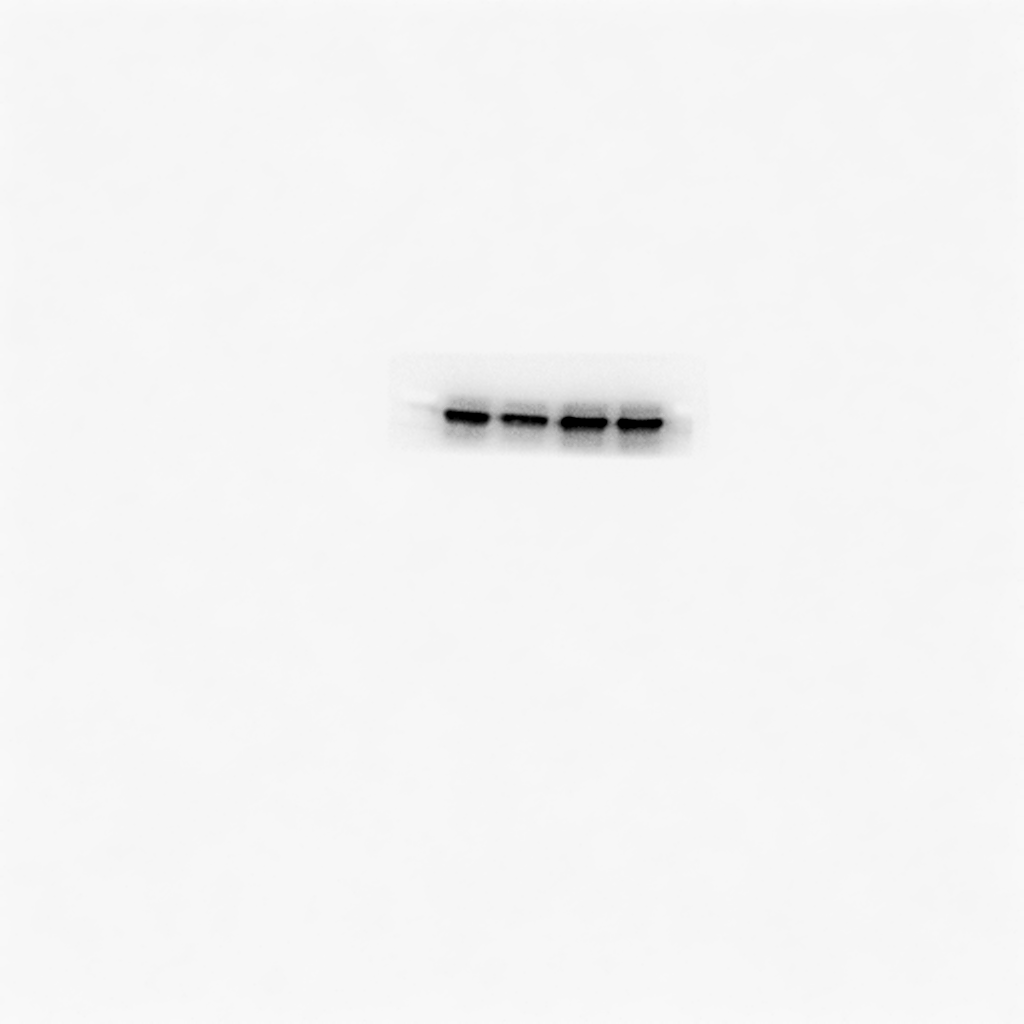

Supplement: Supplemental Information 2 [file peerj-08-9337-s002.zip › Figure 10/Beclin-1/BECLIN 4.2.Tif]

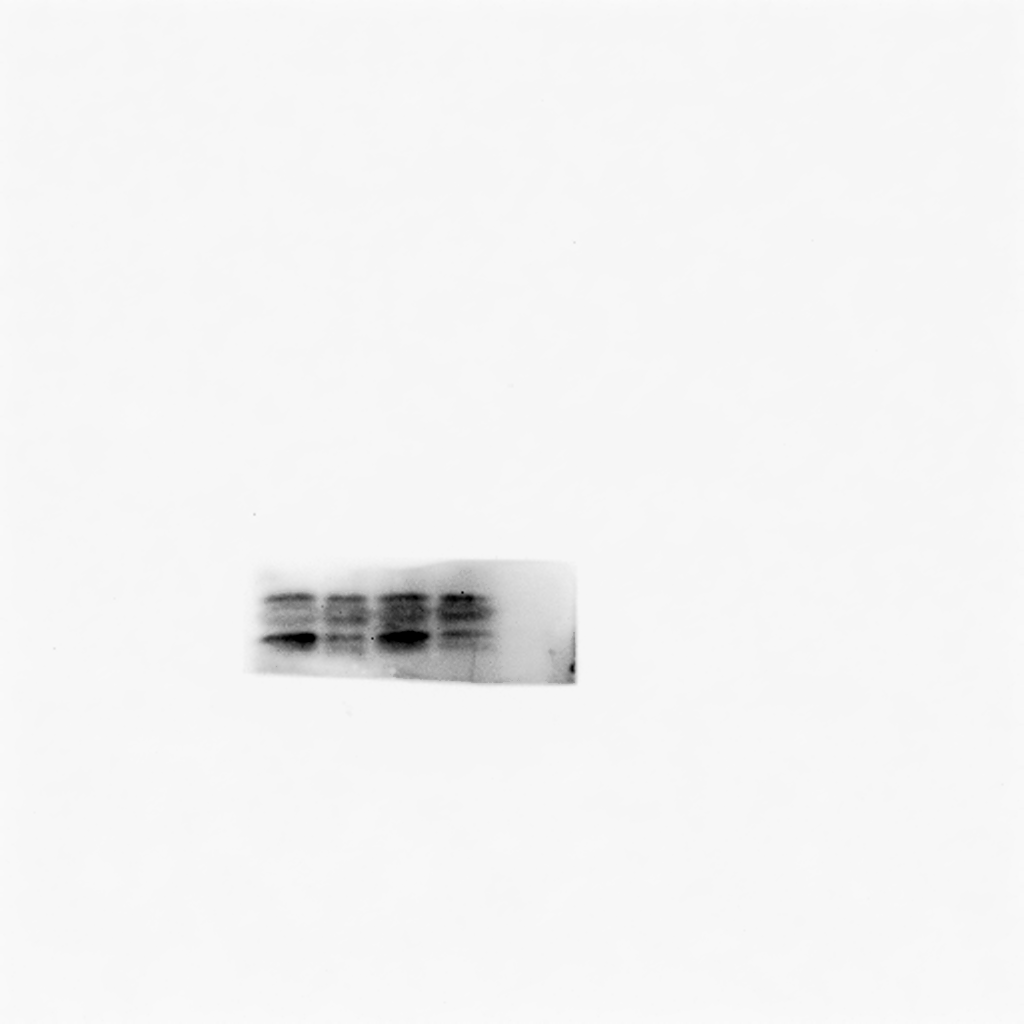

Supplement: Supplemental Information 2 [file peerj-08-9337-s002.zip › Figure 10/LC/LC 3.27.tif]
